# Reactive cutaneous capillary endothelial proliferation predicted the efficacy of camrelizumab in patients with recurrent/metastatic head and neck squamous cell carcinoma

**DOI:** 10.4317/medoral.25919

**Published:** 2023-06-18

**Authors:** Qi Ding, Yang Liu, Houyu Ju, Hao Song, Yuanzhe Xiao, Xiulan Liu, Guoxin Ren, Dongliang Wei

**Affiliations:** 1 Department of Oral and Maxillofacial-Head and Neck Oncology, Ninth People’s Hospital, College of Stomatology, Shanghai Jiao Tong University School of Medicine, Shanghai, China; 2 National Clinical Research Center of Stomatology, Shanghai, China; 3 School of Stomatology, Weifang Medical University, Weifang, China; 4 Department of Radiotherapy, Fengcheng Hospital, Ninth People's Hospital Group, College of Stomatology, Shanghai Jiao Tong University School of Medicine, Shanghai, China

## Abstract

**Background:**

Reactive cutaneous capillary endothelial proliferation (RCCEP), a special adverse event (AE) only observed in patients treated with camrelizumab, was reported to be correlated with the efficacy of camrelizumab in patients with advanced hepatocellular carcinoma. This study to analyze the possible correlation between the occurrence of RCCEP and efficacy of camrelizumab in patients with recurrent/metastatic head and neck squamous cell carcinoma (R/M HNSCC).

**Material and Methods:**

In this study, we retrospectively analyzed the efficacy and RCCEP occurrence of camrelizumab in 58 patients with R/M HNSCC in the Shanghai Ninth People's Hospital affiliated to Shanghai JiaoTong University School of Medicine between January 2019 and June 2022. Kaplan-Meier analysis was used to assess the correlation between the occurrence of RCCEP and the survival of enrolled patients, and COX multifactor analysis was adopted to evaluate associated factors that affected the efficacy of camrelizumab immunotherapy.

**Results:**

A significant correlation between the incidence of RCCEP and a higher objective response rate was observed in this study (*p*=0.008). The occurrence of RCCEP was associated with better median overall survival (17.0 months vs. 8.7 months, *p*<0.0001, HR=5.944, 95% CI:2.097-16.84) and better median progression-free survival (15.1 months vs. 4.0 months, *p*<0.0001, HR=4.329,95% CI:1.683-11.13). In COX multifactor analysis, RCCEP occurrence was also an independent prognostic factor affecting OS and PFS in patients with R/M HNSCC.

**Conclusions:**

The occurrence of RCCEP can show a better prognosis, it could be used as a clinical biomarker to predict the efficacy of camrelizumab treatment.

** Key words:**Reactive cutaneous capillary endothelial proliferation(RCCEP), Recurrent/Metastatic head and neck squamous cell carcinoma (R/M HNSCC), camrelizumab.

## Introduction

Head and neck squamous cell carcinoma is an important cause of death around the world and accounts for a relatively poor prognosis (5-year overall survival rate <50%) (1-3). In recent years, immunotherapy has made breakthroughs and brought new hope to cancer patients with poor prognosis (4-7). In KEYNOTE-048, KEYNOTE-040 and Checkmate-141 study, immunotherapy significantly prolongs median overall survival in patients with recurrent/metastatic head and neck squamous cell carcinoma (R/M HNSCC) (8-11).

Camrelizumab is a highly humanized IgG4 monoclonal antibody that can effectively reduce T cell exhaustion, contribute to sustained anti-tumor effects (12), and demonstrate good efficacy and safety in multiple tumors, such as lung cancer, liver cancer and esophageal cancer, etc. It also showed good curative effect in head and neck squamous cell carcinoma. However, in addition to the common immune related adverse events (irAEs) in immunotherapy, there is also a relatively unique irAE only occurred in patients treated with camrelizumab, namely reactive cutaneous capillary endothelial proliferation (RCCEP) (13-16). It manifests as abnormal proliferation of capillaries usually appears on the surface of the skin, and its morphology is more common in the form of red moles, pearls, and mulberries. It may occurs when camrelizumab reactivates the immune response process, stimulating further secretion of IL-4 and macrophage colony stimulating factor (M-CSF) by Th2 cells, it promotes the differentiation of M2 macrophages, and then promotes the process of abnormal capillary proliferation by releasing VEGF-A (16).

The occurrence of RCCEP has been reported to correlate with the efficacy of camrelizumab in patients with advanced hepatocellular carcinoma (15,17). No relevant research on whether the occurrence of RCCEP is potentially related to its efficacy Camrelizumab in patients with R/M HNSCC. This retrospective study analyzed the occurrence of RCCEP, and the correlation with the efficacy of camrelizumab in patients with R/M HNSCC.

## Material and Methods

- Clinical information and patient inclusion criteria

Between January 2019 and June 2022, 58 patients with R/M HNSCC from Shanghai Ninth People's Hospital affiliated to Shanghai Jiao-Tong University School of Medicine were collected, and the information were obtained from the medical records. The study was approved by the Ethics Committee of Shanghai Ninth People's Hospital. Inclusion criteria for enrolled patients: [1] diagnosis of head and neck squamous cell carcinoma (HNSCC) without indications for surgery or radiotherapy [2] all participants received at least 1 cycle of Camrelizumab immunotherapy combined with chemotherapy; [3] no prior PD-1/PD-L1 therapy; [4] the pathological diagnosis is clearly abnormal capillary hyperplasia rather than hemangioma.

- Therapeutic process and assessments

In this study, patients received Camrelizumab (200mg) in combination with chemotherapeutic agents every 3 weeks. The tumor response assessment was based on computer tomography (CT) or magnetic resonance imaging (MRI) every 2 cycles of treatment according to the Response Evaluation Criteria by Solid Tumors Version 1.1 (RECIST 1.1). The enrolled patients were followed up by telephone, progression-free survival (PFS) and overall survival (OS) were adopted to evaluate the survival outcome. PFS was defined as the time from first treatment to progression or death, whichever occurred first, and OS was defined as the time from initial treatment management to death or loss to follow-up. The grading criteria for RCCEP are defined as follow: grade 1, single maximum diameter ≤10 mm with or without rupture and bleeding; grade 2, single maximum diameter >10 mm with or without rupture and bleeding; grade 3 systemic nodules all over the body, pancytopenic, and may be complicated by skin infections; grade 4, multiple and pancytopenic, life threatening; and grade 5, death.

- Statistical analyses

The cut-off date for our data was June 2022, and all data were analyzed using SPSS Statistics 26.0. Kaplan-Meier survival analysis was used to compare the correlation between the occurrence of RCCEP and long-term outcomes. Chi-square test was used to analyze the correlation between the occurrence of RCCEP and objective response rate (ORR). Cox multivariate analysis was used to evaluate the related factors affecting the efficacy.

## Results

- Baseline characteristics of enrolled patients

Baseline characteristics of 58 patients were shown in Table 1, and patients were aged 18-87 years (average 58.7 years) at diagnosis with a median follow-up of 14 months. 2 patients (3.4%) were lost in follow-up. RCCEP occurred in 39 of 58 patients (67.24%) and in 19 of 58 patients (32.76%) without RCCEP. Of the 39 patients with RCCEP, 13(33.33%) had Grade 1 RCCEP, 24(61.54%) had Grade 2 RCCEP, 1(2.56%) had Grade 3 RCCEP, and 1 (2.56%) had Grade 4 RCCEP. 2 patients stopped the immunotherapy due to RCCEP, and there was no patient with RCCEP leading to death. Among the 39 patients with RCCEP in this study, 10 patients underwent laser therapy and 13 patients had hemostasis therapy, all the patients had complete remission of symptoms.

**Table 1:**
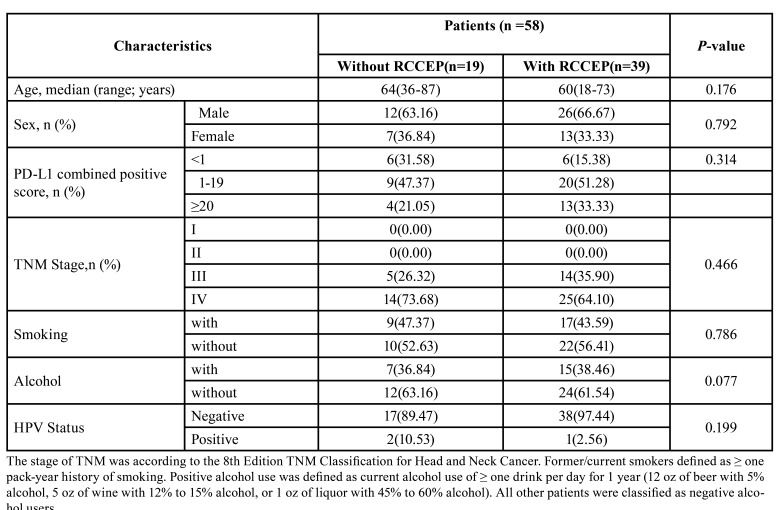
Baseline characteristics of enrolled patients.

- Correlation between RCCEP and efficacy of Camrelizumab

Among the 39 patients with RCCEP, 6 patients achieved complete response (CR) and 13 patients achieved partial response (PR). Among the 19 patients without RCCEP, only 1 patient achieved CR and 1 patient achieved PR. Significantly higher ORR was observed in patients with RCCEP than those without RCCEP (48.72% vs 10.53%, *p*=0.008). The occurrence of RCCEP was also significantly correlated with the short-term efficacy of patients (*p*=0.004) (Table 2).

- RCCEP predicted better prognosis in patients with R/M HNSCC

**Figure 1:**
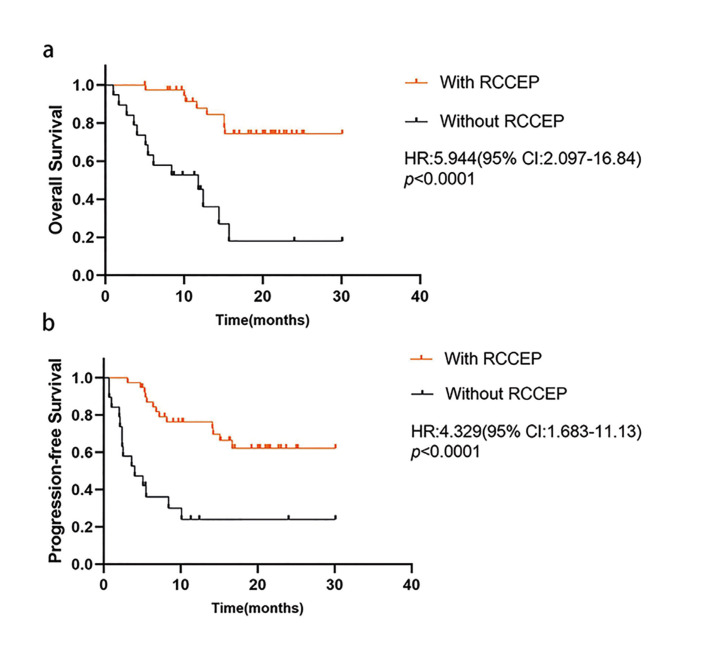
Correlation between the occurrence of RCCEP and clinical outcome. (A) Overall survival (OS) and (B) progression-free survival (PFS) were analyzed between patients with reactive cutaneous capillary endothelial proliferation (RCCEP) and without RCEEP.

Log-rank test analysis showed that patients with RCCEP were significantly associated with better median OS (17.0 months vs 8.7 months, *p*<0.0001, HR=5.944, 95% CI:2.097-16.84) and better median PFS (15.1 months vs 4.0 months, *p*<0.0001, HR=4.329,95% CI:1.683-11.13) (Fig. 1). Cox multivariate regression analysis, which included gender, age, CPS, TNM, occurrence of RCCEP and RCCEP grade in the assessment, suggested that occurrence of RCCEP (*p*<0.0001, HR=7.906, 95% CI 3.054-20.468) were the factors that significantly affected the efficacy of camrelizumab. (Table 3).

**Table 2:**
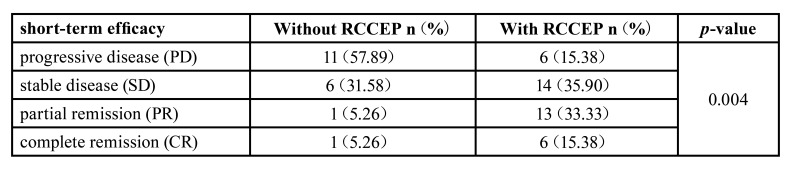
Assessment of short-term efficacy.

**Table 3:**
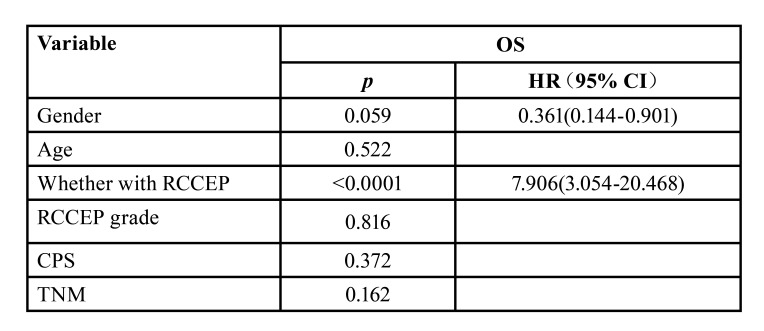
Multivariate analysis of COX in enrolled patients.

## Discussion

Our data is the first report on the relationship between RCCEP and the efficacy of camrelizumab combined with chemotherapy in patients with R/M HNSCC. The results observed that the occurrence of RCCEP was significantly improved the clinical outcome of patients with R/M HNSCC.

PD-1/PD-L1 inhibitors have favorite antitumor activity and tolerate toxicity in patients with R/M HNSCC. RCCEP is a most common and special irAE only observed in camrelizumab treatment. In patients with esophageal and nasopharyngeal cancers, the incidence of RCCEP was reported to be 76.7 % (23/30) and 88% (82/93). In this study, RCCEP was observed in 39 (67.24%) patients (15). Furthermore, several studies have reported that the occurrence of RCCEP is closely related to the efficacy of Camrelizumab treatment (including objective efficacy and survival benefit) (17,18). Three clinical trials of Camrelizumab in lung, liver and esophageal cancer all showed that mOS and mPFS of patients with RCCEP were better than those without RCCEP whenever Camrelizumab was used alone or in combination with chemotherapy (18). In our study, the ORR (48.72% vs 10.53%, *p*=0.008), mOS (17.0 months VS 8.7 months, *p*<0.0001) and mPFS (15.1 months VS 4.0 months, *p*<0.0001) in patients with RCCEP were also significantly better than those without RCCEP. Therefore, RCCEP induced by Camrelizumab can significantly improve the short-term and long-term efficacy of patients with R/M HNSCC. The COX multifactorial regression analysis of this study suggested that the presence of RCCEP were statistically significant, indicating that RCCEP can be used as a clinical biomarker to predict the efficacy of camrelizumab therapy.

In the previous study, common adverse skin reactions, such as rash and pruritus (19,20), require no discontinuation of the camrelizumab administration and could be treated by laser therapy or hemostasis therapy. Among the 39 patients with RCCEP in this study, 10 patients underwent laser therapy and 13 patients had hemostasis therapy, all the patients had complete remission of symptoms. Serious or life-threatening adverse skin reactions are rarely reported (21). There were no deaths due to RCCEP in our study.

However, this study is a single-center retrospective study with insufficient level of evidence-based medicine and relatively insufficient sample size. Therefore, follow-up studies should continue to expand the sample size, extend the follow-up period. Further validate the results of this study by prospective studies with higher evidence-based medical evidence, and that the study has the weaknesses of all retrospective studies.

In conclusion, the occurrence of RCCEP was significantly associated with clinical outcome in patients with R/M HNSCC treated with camrelizumab, and could act as a vital prognostic factor in patients with R/M HNSCC.
